# Autonomous Large-Scale Radon Mapping and Buoyant Plume Modeling Quantify Deep Submarine Groundwater Discharge: A Novel Approach Based on a Self-Sufficient Open Ocean Vehicle

**DOI:** 10.1021/acs.est.3c00786

**Published:** 2023-04-17

**Authors:** Thomas Müller, Jonas Gros, Patrick Leibold, Hajar Al-Balushi, Eric Petermann, Mark Schmidt, Warner Brückmann, Mohammed Al Kindi, Omar S. Al-Abri

**Affiliations:** † GEOMAR Helmholtz Centre for Ocean Research Kiel, RD2/Marine Geosystems, Wischhofstrasse 1-3, D-24148 Kiel, Germany; ‡ Helmholtz Centre for Environmental Research GmbH—UFZ, Permoserstrasse 15, D-04318 Leipzig, Germany; § Ministry of Higher Education Research and Innovation, P.O. Box 82, Ruwi, 112 Muscat, Sultanate of Oman; ∥ Federal Office for Radiation Protection (BfS), Köpenicker Allee 120-130, D-10318 Berlin, Germany; ⊥ Earth Sciences Consultancy Centre, ESSC, P.O. Box 979, P.C. 611, 123 Muscat, Sultanate of Oman; # Mechanical & Industrial Engineering Department, College of Engineering, Sultan Qaboos University, P.O. Box 33, Al-Khoud, 123 Muscat, Sultanate of Oman

**Keywords:** TAMOC plume modeling, autonomous offshore surface measurement, radon, Arabian coastal groundwater, cyclone rainwater infiltration

## Abstract

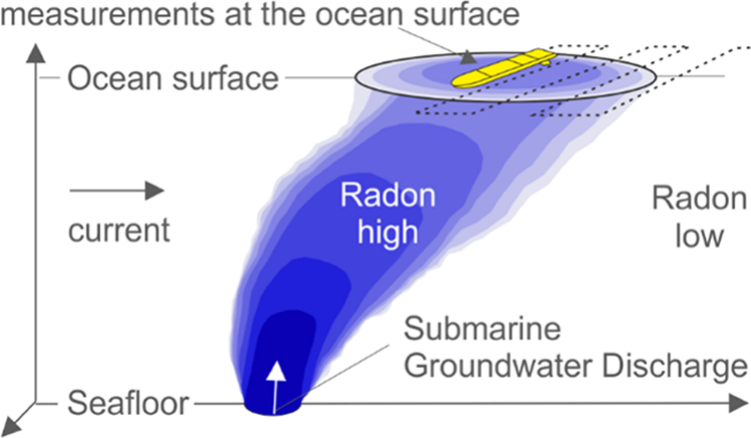

Groundwater discharge into the sea occurs along many coastlines around the world in different geological settings and constitutes an important component of global water and matter budget. Estimates of how much water flows into the sea worldwide vary widely and are largely based on onshore studies and hydrological or hydrogeological modeling. In this study, we propose an approach to quantify a deep submarine groundwater outflow from the seafloor by using autonomously measured ocean surface data, i.e., ^222^Rn as groundwater tracer, in combination with numerical modeling of plume transport. The model and field data suggest that groundwater outflows from a water depth of ∼100 m can reach the sea surface implying that several cubic meters per second of freshwater are discharged into the sea. We postulate an extreme rainfall event 6 months earlier as the likely trigger for the groundwater discharge. This study shows that measurements at the sea surface, which are much easier to conduct than discharge measurements at the seafloor, can be used not only to localize submarine groundwater discharges but, in combination with plume modeling, also to estimate the magnitude of the release flow rate.

## Abstract 2

The quantification of submarine groundwater discharges in water depths >10 m is technologically complex. We report the estimation of the discharge rate of an ∼100 m deep submarine spring based on sea surface radon measurements and a numerical model.

## Introduction

Submarine groundwater discharge (SGD) describes “any and all flow of water on continental margins from the seabed to the coastal ocean, regardless of fluid composition or driving force”.^1^ Hence, two major forms of SGDs can be distinguished: (1) pure freshwater discharge (fresh SGD) from terrestrial aquifers that are connected to the coastal sea driven by a positive hydraulic gradient, and (2) saline, recirculated groundwater (recirculated SGD) that origins from periodical penetration of seawater into the seabed.

SGD occurs on coastlines globally and fresh SGD has been repeatedly proposed as an alternative water resource.^2,3^ Recent estimates state that several tens of km^3^ of freshwater flow into the oceans per year.^4,5^ These studies largely rely on modeled diffuse outflows or water balances for shorelines or catchments. Focused outlets at the seafloor, submarine springs, are found where preferential flow paths exist and groundwater can flow rapidly. Karstic features like conduits or volcanic lava tubes provide these conditions.^6−13^ While it has been shown that submarine springs can have flow rates in the m^3^ s^–1^ range,^14^ only a few direct discharge flow rate measurements exist^7,15^ and especially deep SGD sites have been little described so far.

In the presence of a large volume flow rate and/or large buoyancy flux, a buoyant plume can form which entrains surrounding seawater resulting in dilution of the initial volume of water, typically by several folds. Therefore, the amount of water (mixed with seawater) measured arriving at the surface is expected to represent several times the seafloor discharge,^8^ whereas measurements directly at the outlet require a high level of technical effort or deep-sea technology.

Detection of physical or chemical tracer anomalies at the sea surface such as temperature, salinity, or the radioactive elements radium and radon is useful to identify a submarine fluid discharge from the seafloor.^16−21^ Especially radon (^222^Rn) is an efficient environmental tracer for SGD localization and quantification,^16,22−24^ with most of the existing studies focused on near-shore settings in water depths <10 m. An exception is the Crescent Beach spring 4 km off the Florida Atlantic coast at ∼18 m water depth.^25^ The focus on these near-shore settings is a consequence of easier accessibility of these environments as well as the belief that rapid mixing of SGD with ambient seawater (i.e., dilution) does not allow the detection of a radon signal at the sea surface for offshore settings. However, this belief has not been thoroughly tested for the tracer radon, which can exhibit concentrations in SGD water up to 5 orders of magnitude larger than ambient seawater.^26^

Offshore radon measurements have been carried out at the sea surface, for example, with systems towed by boats^21,27,28^ or as underwater configurations.^29^ However, an important factor limiting the spatial coverage of these approaches is the required logistical effort with regard to manpower and ship time. In the case kayaks or small boats are being used, the area that can be navigated is limited and requires that the discharge location is already well defined. In this study, we acquired new radon field data at the sea surface by using an autonomous offshore surface vehicle in an area for which no SGD had been reported so far. Our data demonstrate that detectable radon anomalies were present at an SGD site located at ∼100 m water depth. We further propose to quantify the release flow rate of the detected SGD by combining autonomously measured ocean surface data, i.e., ^222^Rn as groundwater tracer, with numerical plume transport modeling. The numerical simulation of the outflow is performed by the existing and validated integral plume model Texas A&M Oilspill Calculator (TAMOC),^30^ which describes the trajectory of a single-phase plume of fluid discharged at the seafloor and ascending in the water column. Because some of the parameters could not be sufficiently constrained, we provide a realistic base-case scenario and a likely range of discharge. We further investigate the sensitivity of the estimated flow rate to selected controlling parameters.

## Methods

### Study Site

**Figure 1:**
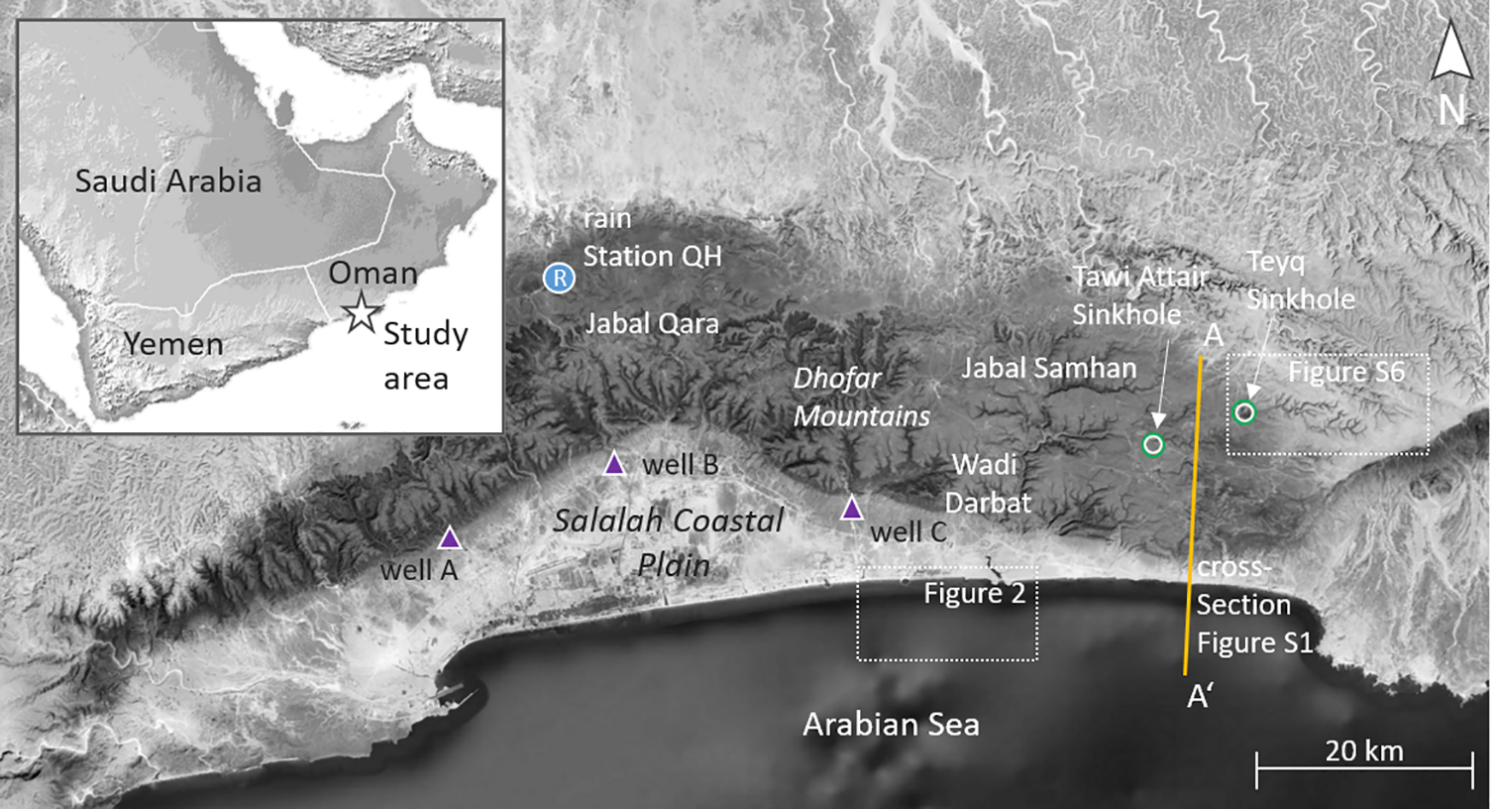
Study area Salalah coastal plain is bordered by the Dhofar Mountains and the Arabian Sea. (A–C) Locations of the groundwater observation wells. Sinkholes Teyq and Tawi Attair are located in the eastern part of the Dhofar Mountains. A geological cross section along the A–A′ segment is available in the Supporting Information (Section S-1). Map imagery reprinted with permission from Earthstar Geographics. Copyright 2022 Earthstar Geographics/Terracolor.

The study area is the eastern edge of the Salalah plain in the southwest of the Sultanate of Oman. Located at the southeast of the Arabian Peninsula (AP), the Dhofar Mountains in the north, east, and west, and the Arabian Sea (AS) in the south are firm borders for the 60 km long arc-shaped coastal plain (Figure 1). The city of Salalah in the center of the plain is the capital of the Dhofar Governorate. Groundwater is the main water source. This reservoir is under pressure, both qualitatively and quantitatively, from increasing extraction, salinization, and anthropogenic pollution.^31−33^ The study area is thus a classic example of the “coastal groundwater squeeze”,^34^ where limited resources are faced with increasing consumption. In the Salalah plain, these limited resources relate to the shallow coastal aquifer (<100 m deep), which is fed by the mountains in the north and is bounded by the ocean in the south. While this shallow reservoir is under pressure, nothing is known of the presumed deep reservoir which can be derived from the geological maps.^35^

The mountain ridge separates the semiarid south (coastal plain and windward side of the mountains) from the arid north (Najd desert). The mountains are essentially formed of two major rock units: basement rocks that were formed during the Proterozoic, and shallow cover sediments that were deposited in the Cretaceous and Cenozoic (Supporting Information Section S-1). The basement rocks comprise igneous and metamorphic rocks including a mixture of mostly gneiss, schist, migmatite, diorite, granite, basalt, and rhyolite.^36^ The shallow cover sediments are principally formed of carbonates, with the Cretaceous carbonates reaching a thickness of 250 m and the Early Cenozoic carbonates of the Umm er Radhuma (UeR) Formation reaching up 600 m. These carbonates include well-bedded and often massively thick units of limestone, which are prone to karstification and weathering. Countless karst features such as caves, sinkholes, dolines, etc. exist.^37^ Some of the caves collapsed and became large sinkholes, as for example the Tawi Attair sinkhole or the Teyq sinkhole.^38^ Other evidences of karst are the (mainly) seasonal springs at the foot of the mountains and submarine karst features.^39,40^ The study area is part of the small fraction of the Arabian Peninsula that receives regular rain from the Indian Summer Monsoon (ISM) which occurs between June and September and brings about 50 mm (coastal plain) to 220 mm (Dhofar Mountains) of precipitation.^41^ In addition, there are also heavy rain events, so-called cyclones, lasting only a few days, occurring infrequently (usually) every 2 to 6 years,^42^ and bringing large amounts of rain to the area, as for example Cyclone Mekunu in May 2018.^43^ Recharge estimates are in the order of 20–30% of the monsoon rainfall in the Dhofar Mountains, resulting in a yearly inflow to the coastal plain of about 40–50 Mm^3^.^32,44^ In 2018, rain and flooding have led to an instant increase in groundwater levels in the coastal shallow aquifer during Mekunu, whereas during the monsoon, when the aquifer is filled by the hinterland mountain aquifer, the increase is delayed and slower (see also Supporting Information Section S-2).

### Field Campaign

Offshore water was screened for SGD by measurement of radon activity concentrations in surface water using an autonomous offshore surface vehicle (Wave Glider SV3–100, Liquid Robotics) serving as sensor platform. The Wave Glider was at sea from December 4th to 7th and December 9th to 14th 2018, surveying an area located 4–8 km offshore to explore possible groundwater discharge from both shallow and deep shelf areas at 50–350 m water depth. The eastern part of the investigated area is the offshore extension of Wadi Darbat, which flows from the Dhofar Mountains into the sea.

**Figure 2:**
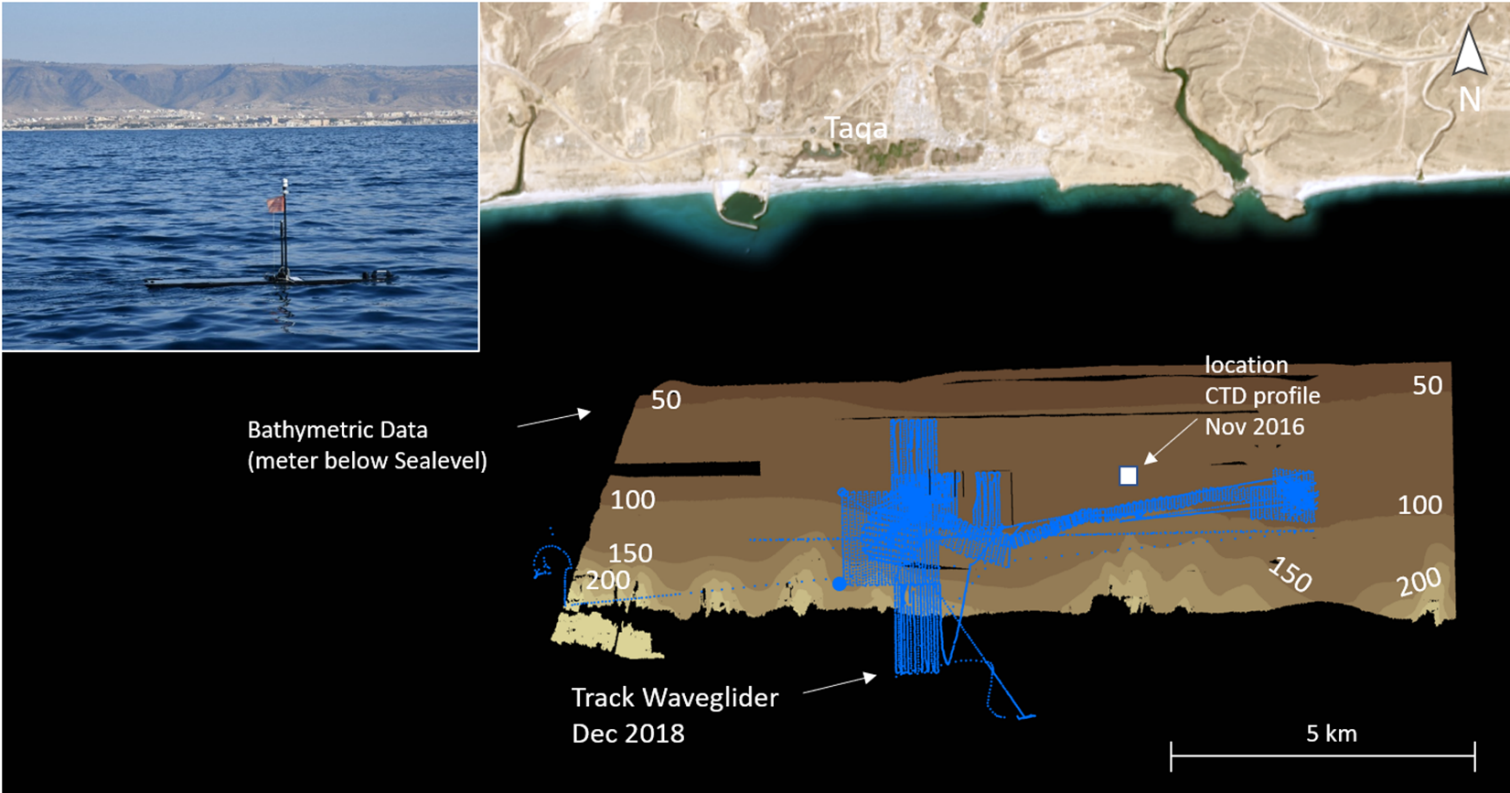
Track covered by the wave glider in December 2018. The photo in the inlet shows the WAVE GLIDER in December 2018 off the coast of Taqa. The location of the CTD cast performed in November 2016 is marked by the white square. Map imagery reprinted with permission from Earthstar Geographics. Copyright 2022 Earthstar Geographics/Terracolor. Photograph by Patrick Leibold.

To optimize detection and tracing of radon anomalies, the survey was conducted with different measurement courses, following mainly the shelf edge where the paleo-shoreline was located during the last glacial maximum, and is now located at about 120 m water depth. All courses were pre-programmed in the form of a sequence of waypoints and were sent from the onshore office to the operating Wave Glider via satellite connection. 24 h piloting enabled full control of recorded data and daily adaption of additional waypoints based on recorded data, actual current direction, and local bathymetry. By using its autonomous obstacle avoidance capabilities, the Wave Glider was able to avoid AIS^1^-enabled targets like commercial coasters or yachts during the survey. The total covered distance was 254 nautical miles with an average speed of 0.8 knots (Figure 2). Basic oceanographic parameters including salinity and water temperature were measured by a Sea-Bird Glider Payload CTD and the GPS track of the Wave Glider was recorded.

To measure ^222^Rn activity concentration a Radon/Thoron Monitor system (RTM1688–2, Sarad GmbH, Germany) was integrated and slightly modified to fit in the payload compartment of the Wave Glider’s float.^45^ A sketch of the general measurement setup is given in Supporting Information Section S-3. The internal gas system of the sensor is coupled to a membrane embedded in a water tank, allowing radon isotopes to pass from seawater into the gas system of the sensor. The tank volume (2.5 l) is continuously exchanged (5 l min^–1^) by a Sea-Bird SBE 5P pump (Sea-Bird Scientific). The ^222^Rn activity concentrations in air measured by the sensor have to be converted into ^222^Rn activity concentrations in seawater. Conversion of measured radon in air to radon in seawater requires consideration of radon partitioning between seawater and air phase. Because the air-to-water volume ratio in the equilibration tube was small, negligible ^222^Rn depletion of the water phase could be assumed.^40^ The respective radon partition coefficient (Oswald’s solubility coefficient) depends on water temperature and water salinity.^46^ Considering the observed salinities of 35.8 and water temperatures of 27–28 °C during the surface water monitoring campaign, a partition coefficient of 0.17 was calculated and was multiplied with “Rn in air” concentration to derive “Rn in water” concentration.^46^

Radon decay measurements were integrated over 5 min (counting cycle), from December 4th at 12:34 pm to December 7th at 03:59 pm and from December 9th at 07:49 am to December 13th at 11:37 pm. Due to spikes in the recorded radon data—probably induced by high-voltage discharges of one out of four measuring chambers of the RTM1688–2 device—the recorded radon data had to be filtered with a specific procedure (see Supporting Information Section S-3). A 10-min response delay of the radon sensor was assumed.^24^ Some basic information on the radon transfer kinetics, based on Petermann and Schuber^24^ is given in the Supporting Information Section S-3. Potential gas ebullition was evaluated to be unlikely to have substantially affected field measurements (Supporting Information Section S-3).

### Bathymetry

Bathymetric maps of 10 m lateral and 1 m vertical grid resolution were analyzed for seafloor morphological features i.e., submarine terraces, depressions, and pockmarks as they are possible indications for submarine groundwater discharge.^47^ The respective maps were derived from multibeam data recorded during a bathymetric campaign in October/November 2016 in the Salalah Bay.^40^ A mobile Kongsberg EM2040C dual-head multibeam echosounder was operated from the Omani research vessel *Al Salt* to map selected areas of the shelf area southeast of Salalah. A frequency range of 200–400 kHz was used for high-resolution mapping at 20–350 m water depth. The maximum depth range of the EM2040C was 400 m. The high-resolution map derived from the multibeam campaign in 2016 was further complemented with bathymetric data obtained from Oman National Hydrographic Office (ONHO) as presented in Figure 2.

### Water Plume Model

Numerical simulations of the SGD outflow were performed with the integral plume model Texas A&M Oilspill (Outflow) Calculator (TAMOC),^30,48^ which describes the trajectory of a single-phase plume of fluid discharged at the seafloor and ascending in the water column. This ascent is principally driven by buoyancy difference or potentially by release velocity, whereas water column stratification is the main obstacle that can prevent seawater from reaching the sea surface.^49^ Consequently, the simulations are driven by the composition of the released fluid (salinity, temperature, radon concentration), the (assumed circular) diameter at the source and the release velocity, the water column profiles of salinity and temperature, and the cross current.

TAMOC includes a multipurpose modeling suite for single and multiphase plumes. The model has been validated for a variety of single-phase plumes and jets with and without cross flow,^30^ and it has further been used and validated for simulating multiphase plumes including gas bubbles and/or liquid hydrocarbon droplets^30,48,50,51^ and glass beads and sediments.^52,53^ The bent plume model of TAMOC predicts the behavior of a plume under cross-flow-dominated conditions. The bent plume model follows a Lagrangian approach to solve for the plume steady-state conditions using a method based on previous works.^54−56^ The bent plume model simulates the effects on the entrainment rate of shear entrainment, forced entrainment from the cross-flow, and buoyancy effects.^30^ Simulations are terminated when the plume either reaches the sea surface or forms an intrusion layer within a stratified water column. TAMOC includes a seawater equation of state, which simulates seawater properties as a function of the local conditions of pressure, temperature, and salinity.^57−59^ TAMOC also enables to track the evolving concentration along the plume trajectory for an arbitrary number of tracers (here radon), which do not affect the plume physics owing to their low concentrations. The bent plume model uses “top-hat” velocity and concentration distributions for internal calculations, which can be converted to the equivalent Gaussian distributions using published literature formulas.^55^ Under the assumption of equal widths of the concentration and velocity distributions, the (average) peak concentration for a Gaussian distribution is defined as twice the top-hat concentration. Integral plume models are able to accurately predict plume concentration profiles downstream of the zone of flow establishment, i.e., at a distance along the plume trajectory (*z*) of >6–10 times the minimum plume diameter (*D*). Within the zone of flow establishment, the inner core of water at the initial concentration and velocity has not yet been “eaten away” by the shear layer.^60^ Therefore, any plume reaching the sea surface at *z*/*D* ≤ 6–10 may present a core having the same radon concentration as the discharged groundwater. The observed pockmark diameters, the water depth, and TAMOC simulations indicated that sea surface measurements were taken outside the zone of flow establishment.

Here, we use TAMOC to provide a range of groundwater discharge rates during the field campaign under simplified assumptions to highlight how the model may be used to estimate water discharge rate under more constrained conditions. We defined a base-case scenario, which broadly agrees with the field observations, using parameter values selected within their uncertainty ranges. Additionally, we performed a sensitivity analysis, where selected parameters were varied over their uncertainty range, which enabled us to define the estimated range of groundwater discharge. This analysis also explored the range of expected values for the least constrained properties and clarified which are the key parameters that control leakage estimates. For the base case, we selected a ^222^Rn activity concentration of 50,000 Bq m^–3^ (maximum concentration observed at three nearby deep onshore wells, in agreement with typical radon concentrations in sediment and meta-sediment aquifers^26^) and a salinity of 13.5 (average of the measurements at the three wells, Table S4). The complete list of selected model parameter values and the justification for the choices made are provided in Supporting Information Section S-4.

## Results and Discussion

### Field Observations

**Figure 3:**
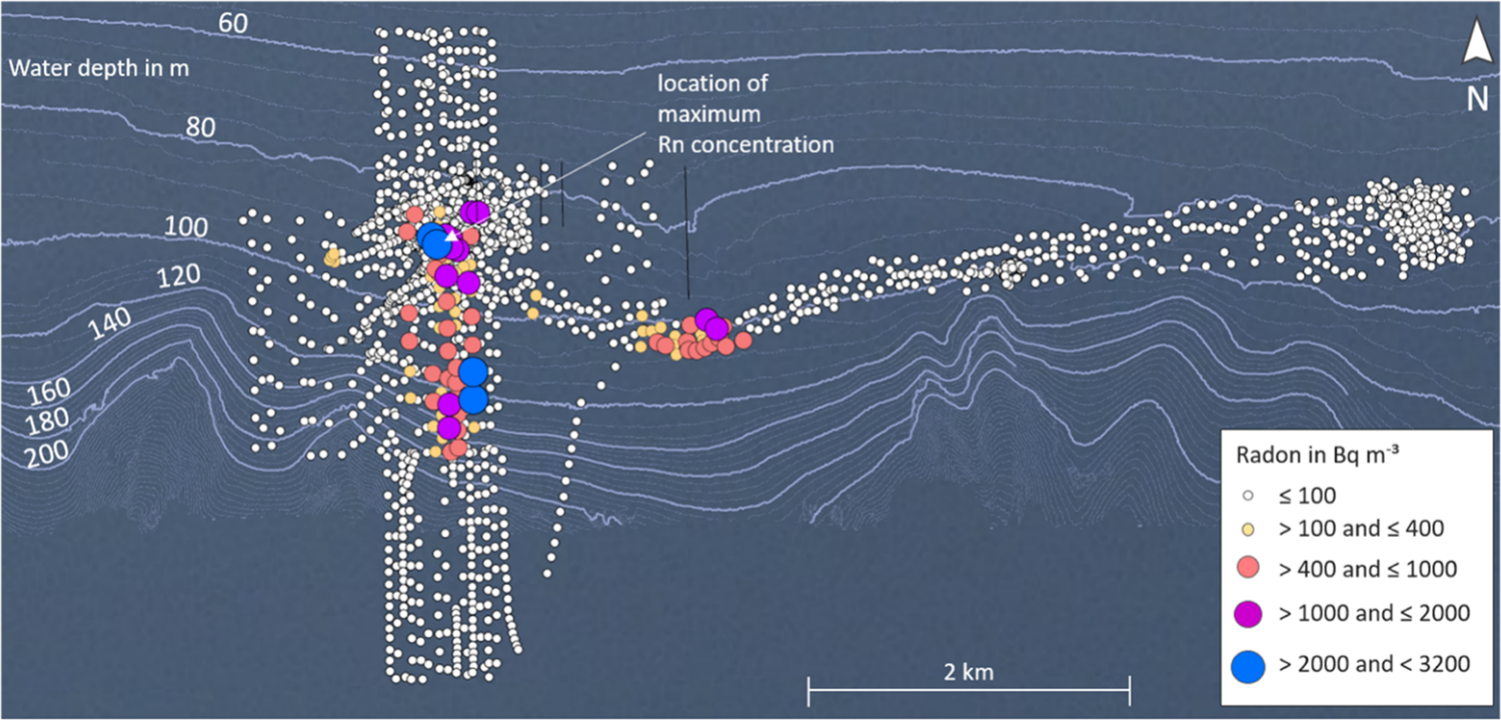
Radon activity concentration in seawater after applying the filtering procedure, overlaid on a bathymetric map of the sampling area.

A total of 1712 radon measurements were collected. Based on the applied filtering procedure the majority of radon data (1265 values) were at seawater background radon concentration (4 Bq m^–3^),^40^ whereas 447 values were above this background concentration with a maximum of 3100 Bq m^–3^. (Supporting Information Section S-3). Three hotspots with increased radon concentrations were identified (Figure 3). The maximum concentration of 3100 Bq m^–3^ was measured on December 11th at 16.98726°N/54.38741°E. This is about 5 km south of Taqa, at 90 m water depth.

Several prominent terraces at about 75/95/115/125 m water depth were observed within the area of investigation, which probably reflect past sea level low-stands.^61,62^ The eastern boundary of the area is characterized by an erosional channel structure which is the prolongation of Wadi Darbat. The southern shelf edge at about 128 m water depth declines toward the upper continental slope area by more than 150 m with a maximum slope angle of 12°. The high-resolution bathymetry acquired in parts of the terraces^40^ indicates small depressions in the seafloor at 110–120 m water depth (Supporting Information Section S-5). A box of 350 × 180 m^2^ located near the area where highest radon values had been measured at the sea surface was analyzed in detail by using 3Dtools of GIS software Global Mapper (Supporting Information Section S-5). The depressions exhibit a circular shape of 3–6 m in diameter and depths of 1–5 m at the center of the depressions (Supporting Information, Section S-5).

### Modeling of Plume Distribution and Discharge Volume Flow Rate

**Figure 4:**
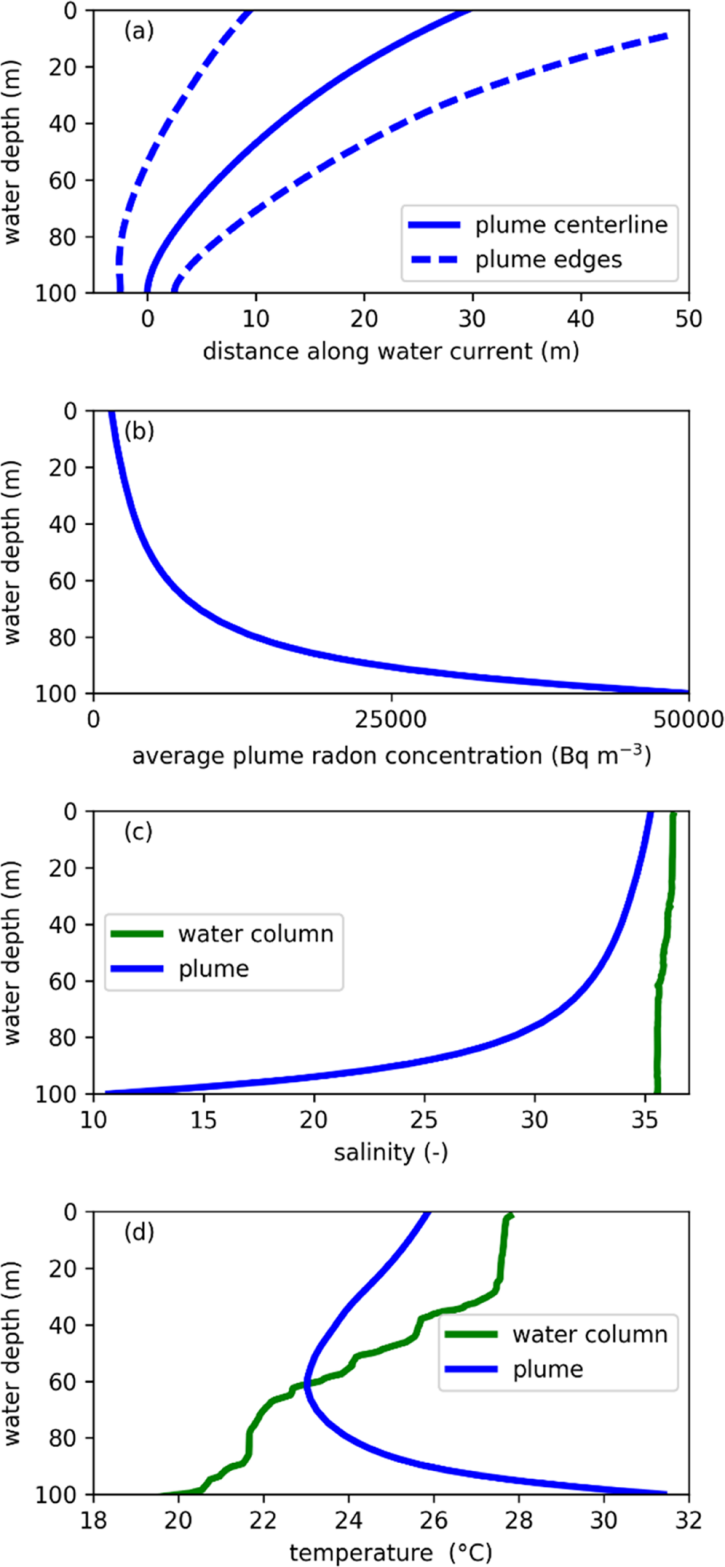
Simulated subsea groundwater discharge for the base case (Supporting Information Section S-4). (a) Plume trajectory (centerline and edges as defined for a top-hat velocity distribution), (b) average radon concentration, (c) water column and plume salinities, and (d) water column and plume temperatures (salinity and temperature profile data from 2016).

The base case simulation (Figure 4a) displays the typical behavior of the simulated plumes for the subsea groundwater discharge for cases where the buoyancy was sufficient for the plume to reach the sea surface, similar to previous observations for single-phase plumes.^30^ The base case simulation (Figure 4) predicts that the water travels within 2 min from the seafloor to the sea surface, entraining a total of 31× its initial water volume. Owing to this dilution, the simulated average plume radon concentration upon reaching the sea surface is 1,500 Bq m^–3^, corresponding to an average peak concentration of 3,100 Bq m^–3^. This value is 3 orders of magnitude larger than in situ measurement of the background radon concentration in seawater (4 Bq m^–3^), which confirms the fitness of radon as a tracer of the released groundwater. The simulated plume salinity reaches a value of 35.3 at the sea surface, which is ∼1 unit lower than the sea surface water salinity of the selected CTD profile. This is larger than the observed salinity variability of <0.1 for all measurements collected by the wave glider during the field measurements. This discrepancy might be explained by the low CTD sampling rate (0.2 min^–1^ or 130 m interval), which we postulate led to measurements missing the locations with the largest deviations to baseline, as the sampling interval was likely larger than the plume width (Figure 4a). Salinity and temperature sensors have very fast response times such that high deviations to baseline can easily be missed when using a low sampling rate; we therefore advise to record these data at high sampling rates in future studies. By contrast, the long response time of the radon sensor ensures that anomalies are detected at the selected data acquisition rate.

Salinity and temperature profile data which were used for simulations originate from the same area and the same season but from a different year (2016) than the sea surface measurements due to the lack of salinity and temperature profile data for 2018. This circumstance contributes to uncertainty in the predicted salinity and temperature of the plume water reaching the sea surface, and a CTD profile should be acquired in close temporal and spatial proximity of the wave glider deployment for future applications of the approach.

**Figure 5:**
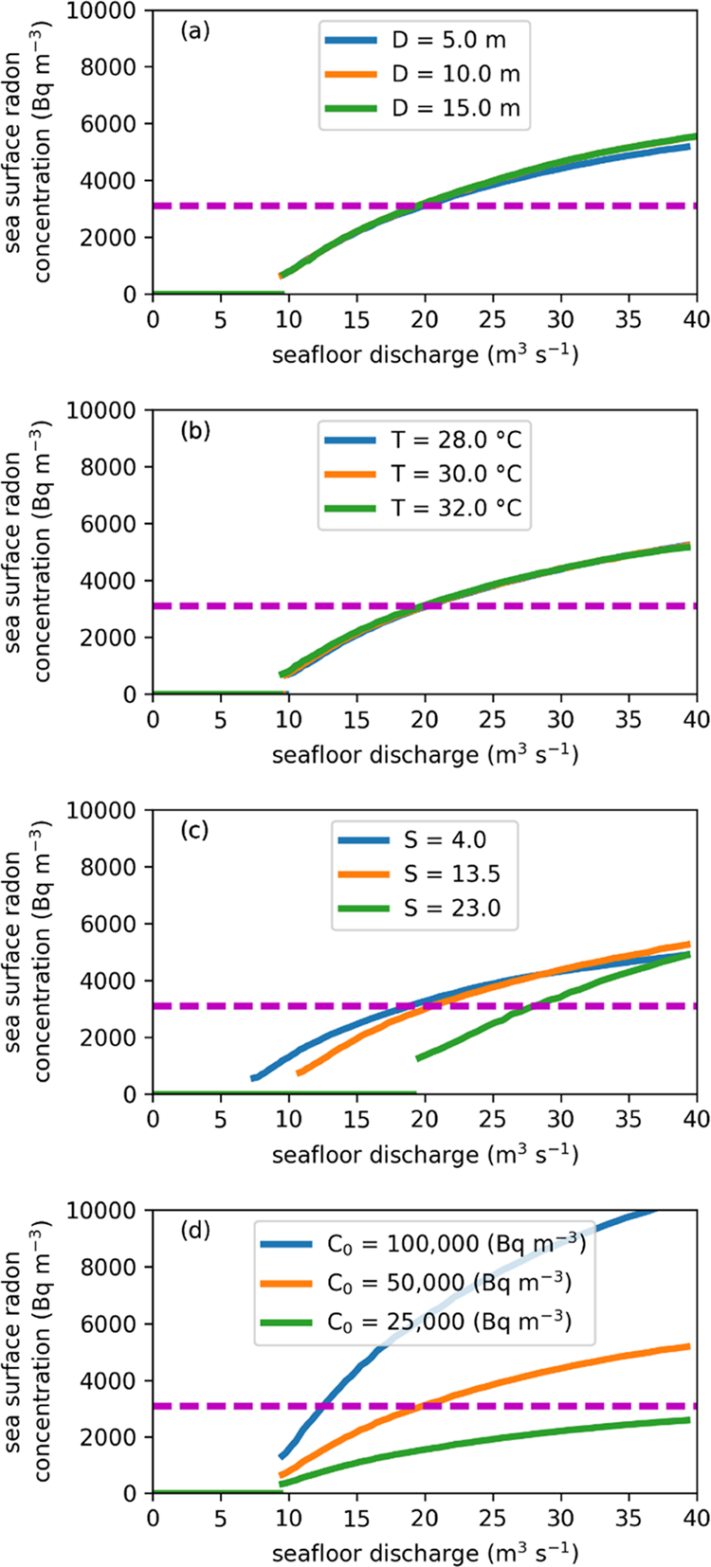
Sensitivity analysis of the simulated sea surface average peak radon concentration as a function of seafloor discharge for varied (a) seafloor source diameter (*D*), (b) discharged water temperature (*T*), (c) discharged water salinity (*S*), and (d) discharged water radon concentration (*C*_0_). Solid green, orange, and blue lines present different scenarios. The purple dashed lines indicate the maximum radon concentration measured at the sea surface (3,100 Bq m^–3^) is displayed (purple dashed line). The solid lines do not extend past a water velocity of 2.0 m s^–1^ upon exiting the seafloor (Supporting Information Section S-4).

A volume flow rate of discharged water of >7.4–19.6 m^3^ s^–1^ is necessary for the plume to have overcome the water column stratification and have reached the sea surface from a 100 m depth seafloor for the assumed 0.27 m s^–1^ water current and temperature and salinity profiles, according to the TAMOC simulations (Figure 5), which defines the minimum estimated seafloor discharge. If assuming a seafloor discharge of water containing 50,000 Bq m^–3^ of radon, a discharge of 19–28 m^3^ s^–1^ at the seafloor would lead to an average peak radon concentration of 3,100 Bq m^–3^ at the sea surface. Among the investigated parameters, the initial radon concentration in the discharged water together with the average peak radon concentration measured at the sea surface appears to be the main controlling parameters for estimating the groundwater discharge. The large uncertainty range of the salinity of the discharged water, which acts as the main source of buoyancy for the plume, also affects the predicted radon concentrations at the sea surface. An additional finding is that for a seafloor discharge of water containing 50,000 Bq m^–3^ of radon, any plume reaching the sea surface is predicted to be easily detectable with the chosen instrumentation, with average peak concentrations ≥440 Bq m^–3^, or ≥100× larger than the ambient seawater.

### Source of the Discharged Water

**Figure 6:**
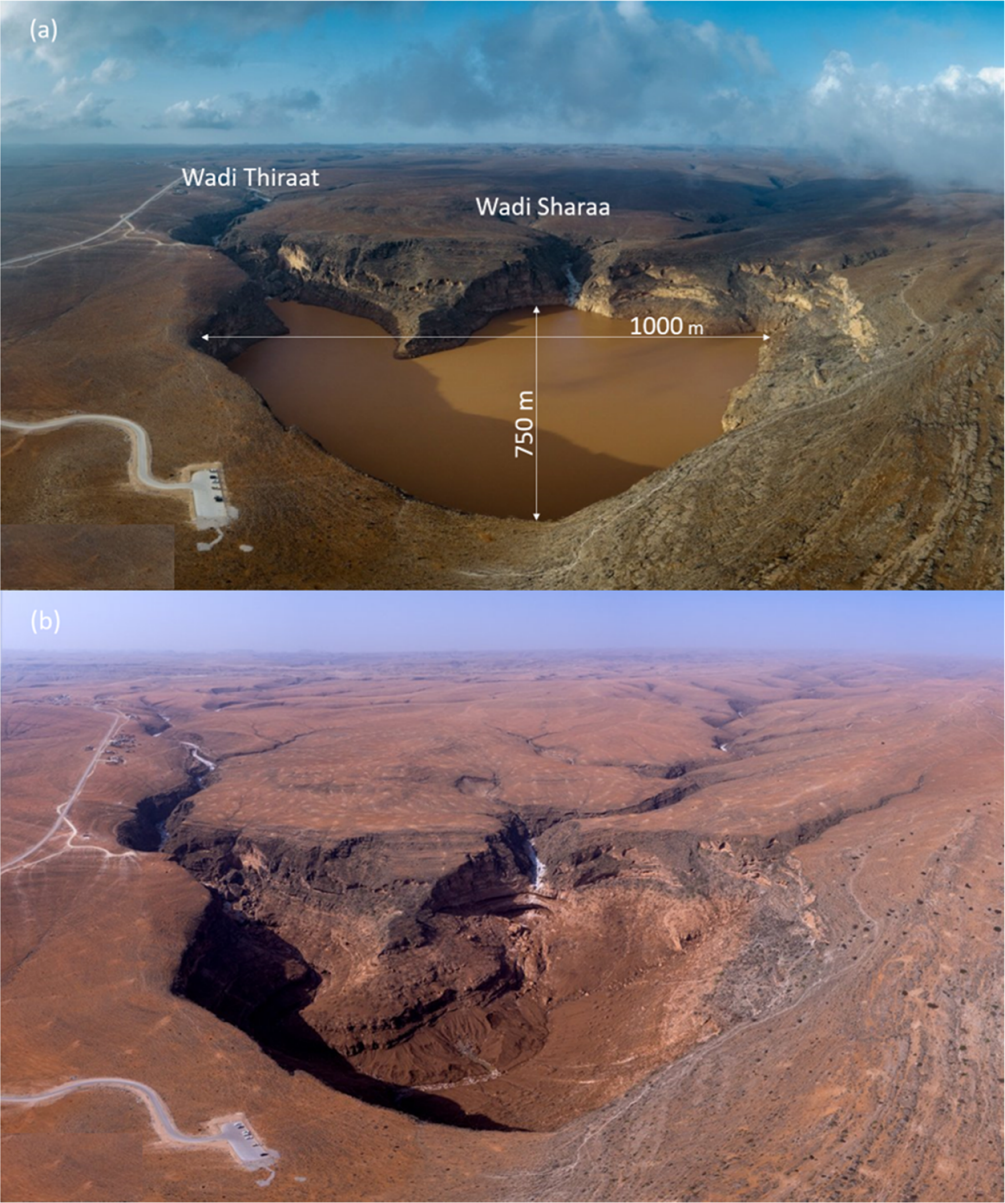
Aerial view of the Teyq Sinkhole. (a) Filled sinkhole on May 30, 2018. (b) Empty sinkhole on June 1, 2018 (photographed by Ahmed Altoqi; https://twitter.com/AhmedAltoqi).

The year 2018 was an exceptionally wet year for southern Dhofar. In addition to the annual monsoon three cyclones occurred: “Sagar” and “Mekunu” in May and “Luban” in October.^63−65^ During Sagar little rainfall was observed in the coastal plain (a single own measurement via funnel resulted in 9 mm on May 16th), whereas up to 145 mm fell during Luban from October 13th to 15th.^65^ Mekunu brought much heavier rainfall. Station measurements exceeding 600 mm have been recorded from May 24 to 27 for Salalah^64^ and 745 mm at station QH^43^ in the Dhofar Mountains (see Figure 1 for location) while maximum amounts in the order of 3,000 mm are given for the Salalah area.^64^ Large wadi floods both north and south of the Dhofar Mountains occurred, with parts of the central coastal plain under water for several days and 6.4 × 10^6^ m^3^ of water collected by the Sahalnawt dam (protection dam for Salalah city and airport).^64^ The Teyq Sinkhole was filled during Cyclone Mekunu. The sinkhole has a maximum depth of about 280 m, and extends 1000 m across its *W*–*E* axis and 750 m across its *N*–*S* axis,^66^ offering a huge water storage capacity. Surface runoff collected in Wadi Sharaa, which comes from the east, and Wadi Thiraat, which comes from the north filled the sinkhole. Videos from May 28 and 29 show that the sinkhole was at least half full.^67,68^ While the sinkhole was still filled to a good degree on May 30th (Figure 6a), by the afternoon of June 1st all water had drained out (Figure 6b).^69^ Since the hole emptied within <3 d, we know that most of the water ran off underground since evaporation was negligible. How much water has collected in the sinkhole is unclear. Both wadis together have a surface catchment area of 50.2 km^2^ (Supporting Information Section S-6). Precipitation data for stations nearby are not available. With the 745 mm of station QH, which is 55 km west of the sinkhole (see Figure 1), the accumulated volume would be 35 × 10^6^ m^3^.

However, the observed filling of the sinkhole on May 28 and 29 suggests that a larger amount of water has accumulated. Considering the sinkhole as a cylinder with a diameter of 500 m, and a height of 200 m gives a total volume of 160 × 10^6^ m^3^. Filled to 50% the volume is 80 × 10^6^ m^3^. This would require 1,600 mm precipitation, which is in the order of the maximum amount cited above.^64^

With the above estimated discharge of 19–28 m^3^ s^–1^, a single submarine spring could pour water for 14–21 d (assuming a volume of 35 × 10^6^ m^3^) or 33–49 d (assuming a volume of 80 × 10^6^ m^3^)—if only the volume of Teyq is considered. There are other smaller and larger structures—for example, the Tawi Attair sinkhole with its 21 km^2^ catchment—which also allows the accumulation and drainage of large volumes of water to the underground. Since rainfall occurred along the whole, approx. 400 km long coast of Dhofar,^43^ it can be assumed that in the Dhofar Mountains additional water was available, probably in the order of few hundred million cubic meters. While this water first had to infiltrate and pass the deeper geologic formations, we postulate that high infiltration rates during Mekunu caused SGDs offshore Taqa 180 d later. The observed characteristics (flow rate above 10 m^3^ s^–1^, and water depth up to 150 m) have been described for submarine springs.^14^

What happens between inflow and outflow remains speculative, at least for the deep reservoir. There are 6 months between the infiltration (beginning of June) and the measurement campaign (beginning of December). While the filling time is known, it is not known since when the submarine spring had been active. The karst structures enable large amounts of water to be quickly absorbed on the one hand and point discharge at the seafloor on the other hand. An explanation for a delayed drainage could be the geology of the basement through which the water has to pass. A possible explanation for the long residence time in the hydrogeologic system could be that the metamorphic and igneous rocks of Proterozoic and Cretaceous age are less permeable than the limestones of the Dhofar Mountains, and act like a bottleneck for the water. The pockmarks observed at the seafloor allow then a fast outflow once the less permeable formations are passed.

The SGD radon endmember depends only on the “radon potential” of the aquifer rock material in which the groundwater was during the last days before discharge. This is a consequence of the short half-life of radon (∼4 d). The radon signature of the groundwater is continually overwritten. Considering the 4 d half-life, it means that after 20 d (5 half-lives) 97% of the radon that was originally in the water decayed.

The modeling has shown that for reaching the average peak concentration of 3,100 Bq m^–3^ at the sea surface, a seafloor discharge of water containing 50,000 Bq m^–3^ of radon is necessary. Thus, the water must have been in contact with a geological formation which has a radon potential that allows such values in the water. There are no radon data on the formations in the study area. However, radon concentrations in groundwater from sediment, meta-sediment or granitic aquifers, which are all found in the study area, show a wide range of possible values ranging typically from 10,000 to over 1,000,000 Bq m^–3^.^15^

### Outlook

This study presents an approach to detect and quantify SGDs based on field observations (^222^Rn, T, S) at the sea surface and cheap, broadly available water column measurements (CTD profile and bathymetry). Under simplified assumptions, the TAMOC model proved able to simulate the SGD and provide an estimate of the range of possible groundwater discharge rates. This implies that when using suitable technology, measurements of radon at the sea surface can be used not only to identify SGDs but also to estimate the magnitude of the flow. This is particularly promising because sea surface technologies are usually easier and cheaper to deploy to survey large areas and are therefore likely to be retained for SGD detection. Consequently, expensive and more complex additional equipment does not need to be deployed at the seafloor if the quantification of flow rate can be based chiefly on the same technology used for detection. Further, measurements of other environmental tracers (such as radium isotopes, salinity/conductivity, and temperature) in sea surface water and groundwater would complement the current approach and have the potential to further extend the understanding of the investigated system.

The cyclonic storm Mekunu was identified as one likely driver for the groundwater discharge. However, what happens precisely between infiltration on land and discharge subsea remains unknown. A land-based tracer test, which was already planned in the past but has never been implemented, could reveal more insight into the dynamics of the Teyq karstic system and its relation to SGD. The observed occurrence—at least three strong rainfall events in the last 9 years filled up the Teyq sinkhole—indicates that this could be feasible within reasonable timescales.

Although the presented data and interpretations suggest that a lot of freshwater, possibly in the range from a few tens to hundreds of millions of cubic meters, is temporally available, we remain currently far from the ability to use it for water supply onshore. The local setting would be favorable: semiarid area, water scarcity, short distance of the SGD outlet to the coast. However, the event appears to be stochastic in its occurrence in space and time, to have limited predictability, and to bring extremely large amounts of water, which are available in a very short time. There are also technological obstacles, including the technological effort to collect the water and to transport it onshore, and the necessity to include a desalination treatment.

The tools used in the present study identified and made a first estimate of the discharge rate of a previously unknown submarine spring―without expensive exploration or deep-sea technology. Our results show the suitability of radon as a powerful tracer not only for shallow, near-coastal but also for deep, offshore SGD in specific settings (focused discharge, ascent of buoyant plume present, large land-ocean gradient). Autonomous detection systems may allow to screen large areas with low amount of work. A systematic screening along shorelines with preferable hydrogeological settings can contribute exploring SGD to the coastal Ocean.

## Supporting Information Available

Geologic cross-section Line A-A'; groundwater level development for three wells in the Salalah coastal plain; radon transfer kinetics and radon data correction; model parameters used in the TAMOC simulations; bathymetry information; and catchment area of the Teyq sinkhole (PDF)

## Footnotes

1 automatic identification system
