# Supplementary material for: Autonomous Large-Scale Radon Mapping and Buoyant Plume Modeling Quantify Deep Submarine Groundwater Discharge: A Novel Approach Based on a Self-Sufficient Open Ocean Vehicle
Source: Environ Sci Technol. 2023 Apr 17;57(16):6540–9. doi: 10.1021/acs.est.3c00786 (PMC10134489; doi:10.1021/acs.est.3c00786)
Supplement: Supplementary file 1 — es3c00786_si_001.pdf [file es3c00786_si_001.pdf]

Supplementary Information for:

Autonomous large-scale radon mapping and buoyant plume modelling quantify deep submarine groundwater discharge - a novel approach based on a self-sufficient open ocean vehicle

Thomas Müller, [*,†,‡] *Jonas Gros,*[†] *Patrick Leibold,*[†] *Hajar Al-Balushi,*[§] *Eric Petermann,*[‖] *Mark Schmidt,*[†] *Warner Brückmann,*[†] *Mohammed Al Kindi,*[⊥] *and Omar S. Al-Abri*[=]

[†] GEOMAR Helmholtz Centre for Ocean Research Kiel, RD2/Marine Geosystems

Wischhofstrasse 1-3, D-24148 Kiel, Germany

[‡] Helmholtz Centre for Environmental Research GmbH – UFZ

Permoserstrasse 15, D-04318 Leipzig, Germany

[§] Ministry of Higher Education Research and Innovation, Muscat, Oman

P.O. Box 82 Ruwi, 112 Muscat, Sultanate of Oman

[‖] Federal Office for Radiation Protection (BfS), Berlin, Germany

Köpenicker Allee 120-130, D-10318 Berlin, Germany

[⊥] Earth Sciences Consultancy Centre, ESSC, Muscat, Oman

P.O. Box 979, P.C. 611, 123 Muscat, Sultanate of Oman

= Sultan Qaboos University, Mechanical & Industrial Engineering Department, College of Engineering

P.O. Box 33, P.C. 123 Al-Khoud Muscat, Sultanate of Oman

Contents of this file: Pages S1 - S17

Figure S1 - S6

Tables S4, S5

This document provides:

- geological cross-section Line A-A' (section S-1)
- groundwater level development for three wells in the Salalah coastal plain (section S-2)
- radon data transfer kinetics and radon data correction (section S-3)
- model parameters used in the TAMOC simulations (section S-4)
- Bathymetry information (section S-5)
- catchment area of the Teyq sinkhole (section S-6)

S-1 Geological cross-section

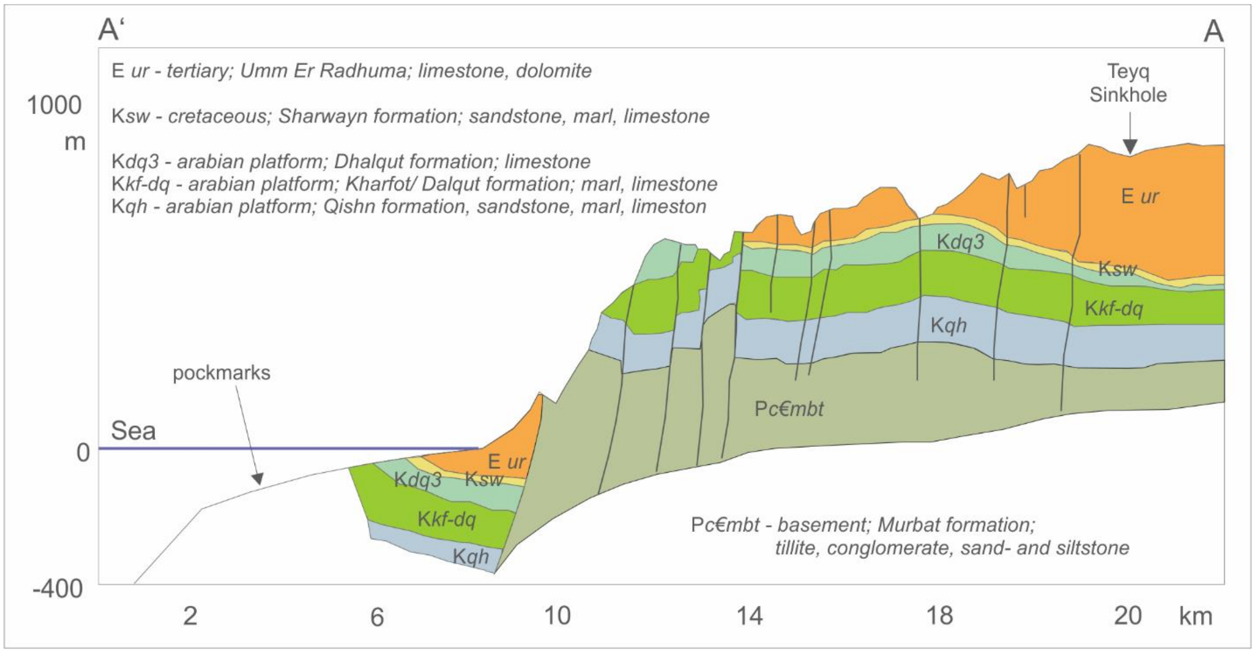

Figure S 1. The Dhofar mountains represent a very complex geologic structure with a multitude of faults and discontinuities. In the eastern part of the plain cretaceous and Paleozoic deposits wedge out while the coastal strip and the coastal aquifer is very narrow. The position of the sinkhole and the pockmarks are projected onto the cross-section. See Figure 1 for location. Sketch modified from Platel et al.[1]

S-2 Water level groundwater wells

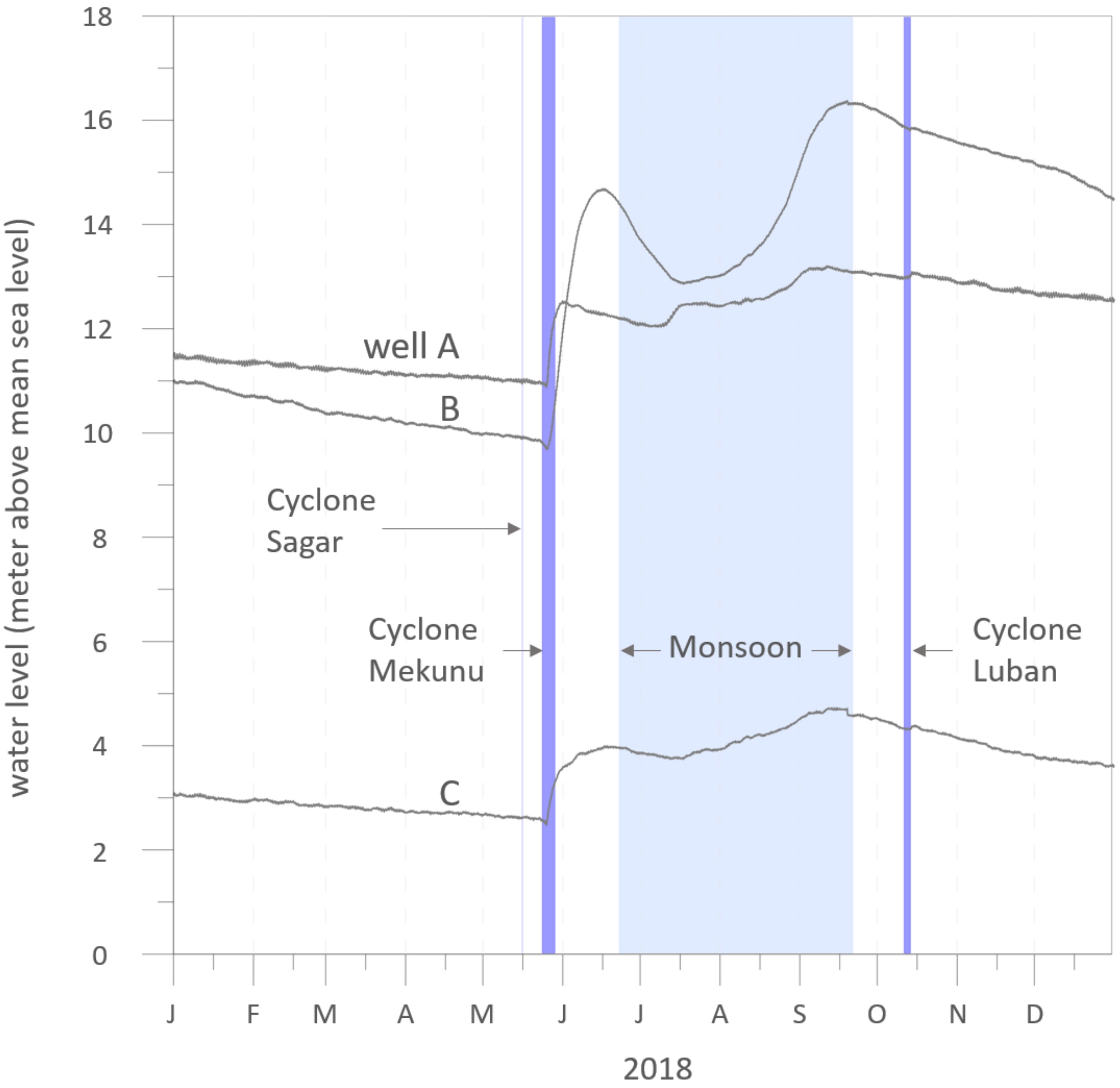

Figure S 2. Groundwater level at 3 wells in the Salalah coastal plain for the year 2018. Well A in the west, B in the central part, and C in the east of the plain. See Figure 1 for location of the wells.

S-3 Measurement setup, Radon transfer kinetics, Radon data filtering procedure, and gas ebullition

Measurement setup

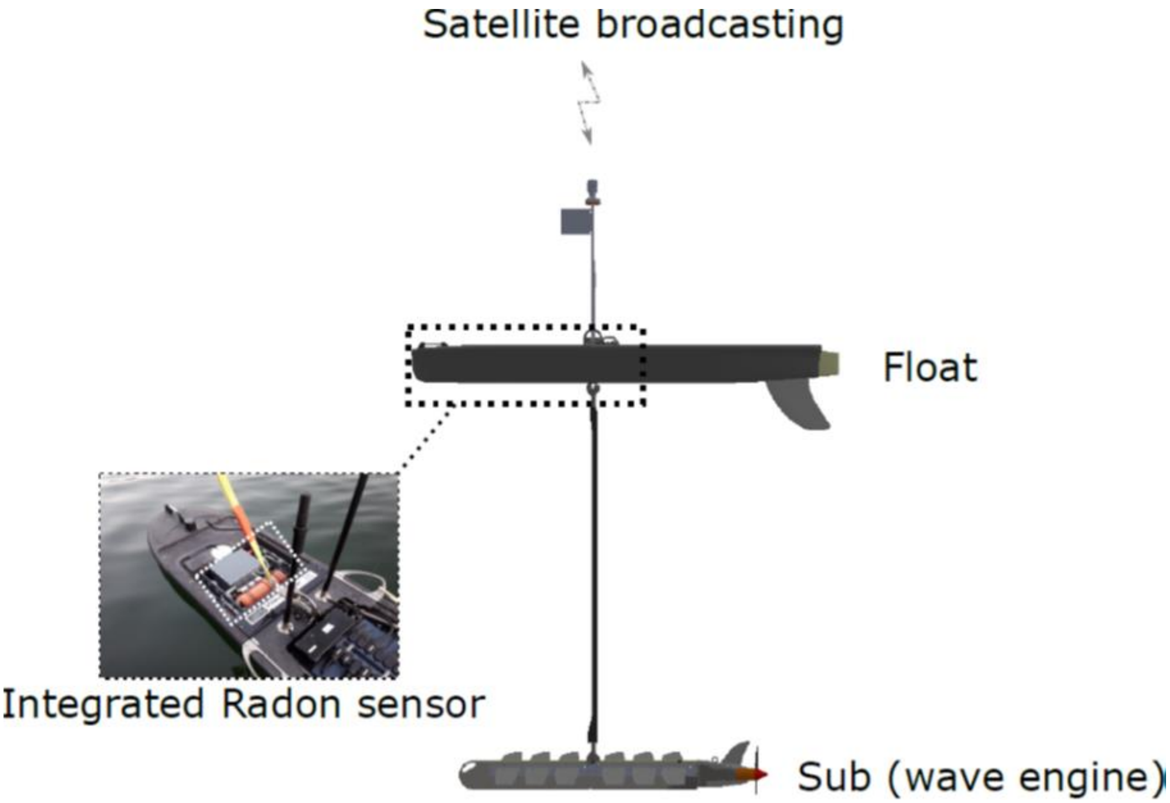

Figure S 3-1. General setup of the Wave Glider SV3 measurement system. The integrated Radon sensor payload is marked by the dotted white rectangle. Scheme reprinted with permission from Liquid Robotics. Photograph by Patrick Leibold.

Radon transfer kinetics

The explanations and figures shown below refer to the results of Petermann et al.[2]

The radon transfer between water and air depends on many factors, including:

- type of exchange unit (e.g., membrane, spray unit)
- water pumping rate
- air flow rate through the measurement chamber
- volume of the measurement chamber

Experiments have shown that the delay is caused by two processes: a kinetic delay and a decay delay (Figure S 3-2). The black line represents true radon-in-water anomaly, red is the radon-in-air (corrected for partitioning) in the measuring chamber (delay caused by gas transfer kinetics) and green the system measurements (it measures Po-218). The radon transfer into the system is caused by the kinetic delay (from black to red line), the decay of radon into Po-218 relates to the decay delay (from red to green line). In the observed signal we see a response delay and a smoothing of the signal relative to the input signal.

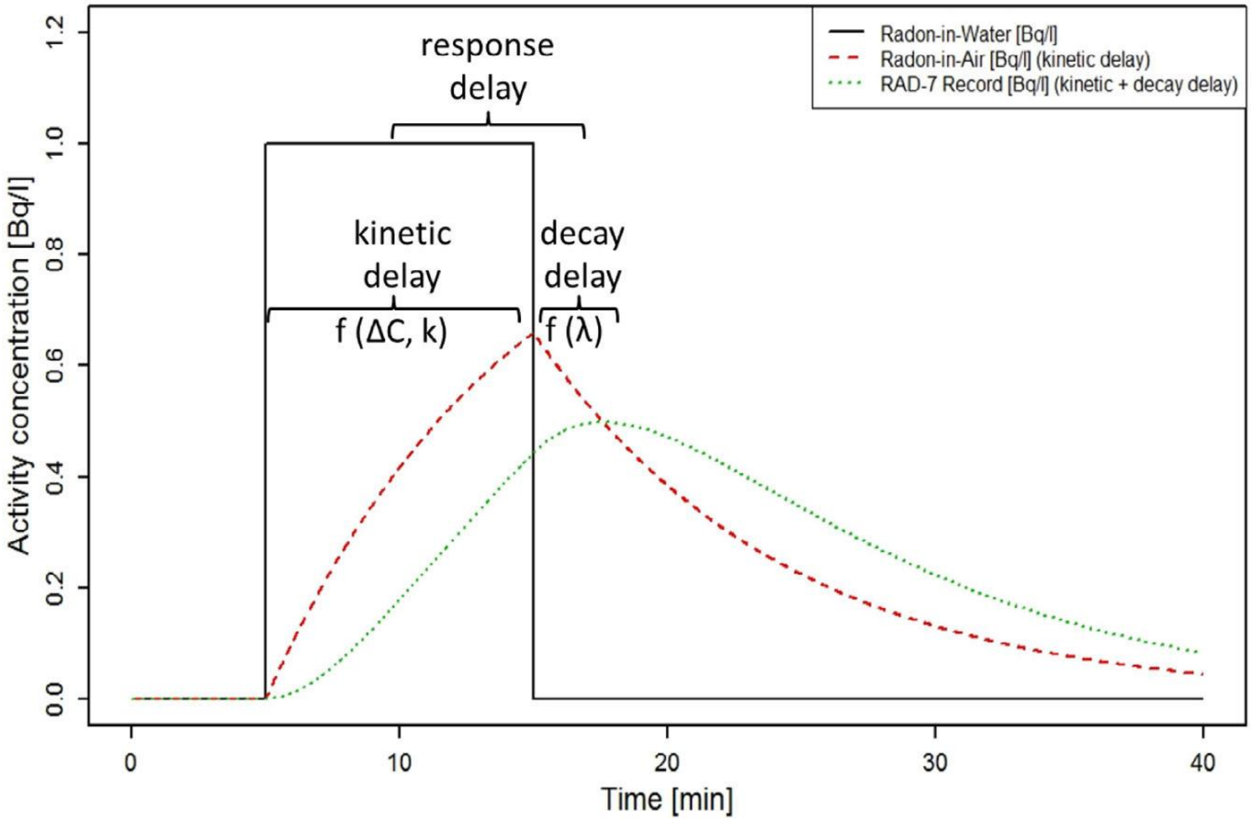

Figure S 3-2. Response delay is made up of kinetic delay and decay delay. Figure is taken from Petermann et al.[2] With kind permission of the European Physical Journal (EPJ). European Physical Journal Special Topics, 224, 2015, page 700, Eric Petermann and Michael Schubert, (c) EDP Sciences, Springer-Verlag 2015.

For a more complex signal the input and the measurement record are shown in Figure S 3-3. Both figures show that radon transfer kinetics are not negligible for radon detectors. Transfer kinetics are the dominant factor causing the response delay. To reach an equilibrium between input and observation it takes usually 30-40 min. The set-up used in the present study (exchange via membrane, flow rate 2.5 L/min, etc.) is similar to the one used in the experiment. Consequently, it is reasonable to assume that the response delay is similar as well.

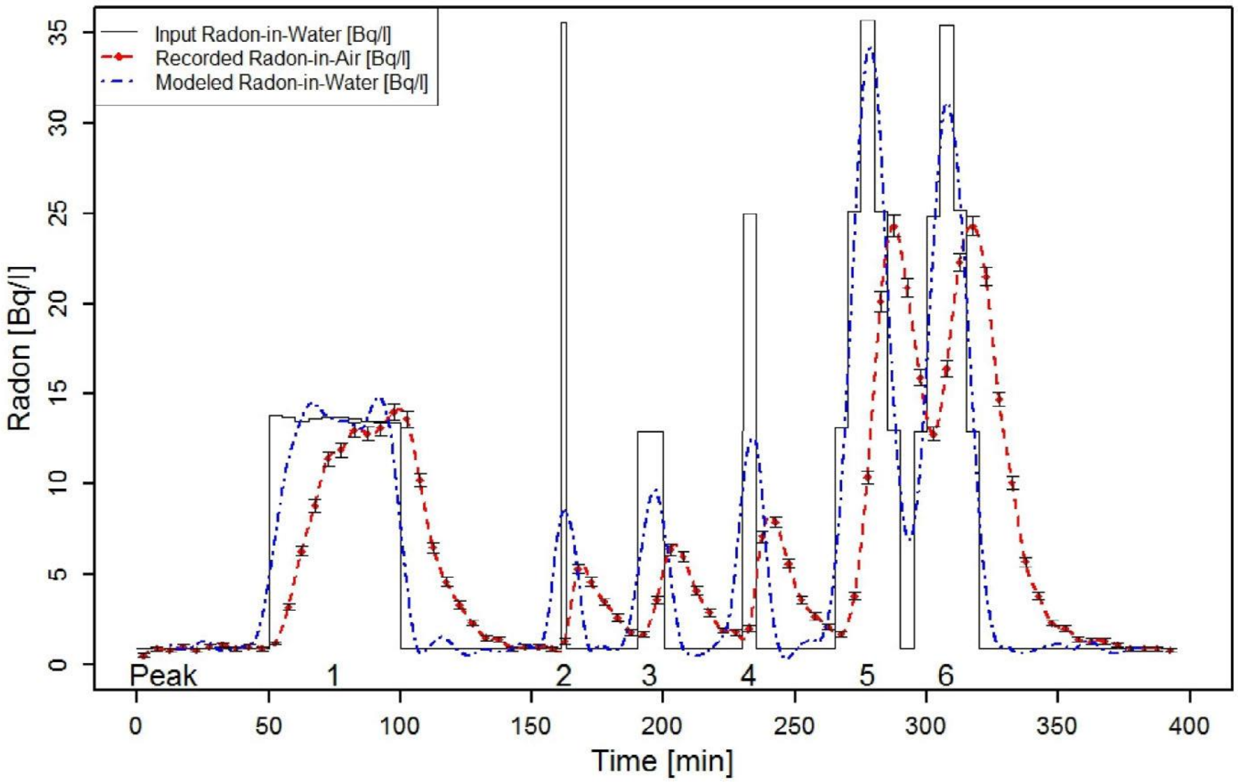

Figure S 3-3. Radon-in-water concentrations (input signal) compared to the recorded radon-in-air concentrations and the modelled radon-in-water concentrations. Figure is taken from Petermann et al.[2] With kind permission of the European Physical Journal (EPJ). European Physical Journal Special Topics, 224, 2015, page 700, Eric Petermann and Michael Schubert, (c) EDP Sciences, Springer-Verlag 2015.

Filtering procedure for the radon data

A data filtering procedure had to be applied as recorded ^{222}Rn-data was partly corrupted by electronic spikes, which where possibly caused by high-voltage sparks in one detection unit of the radon sensor unit RTM-1688.

To apply sound data filters a 3-step algorithm of if-then equations was used to filter raw data:

1. If a given ^{222}Rn activity concentration in air is below the measured local background, i.e. N_i <24 Bq m^{-3}, then set the ^{222}Rn activity concentration to 24 Bq m^{-3}; ^{222}Rn activity

concentrations were recorded in "fast mode" with 5 min sampling time (however, the local background ^{222}Rn activity concentration of 24 Bq m^{-3} was separately recorded with a 15-min sampling time).

2. If the ^{222}Rn activity concentration in air $N_i > 15 x N_{i-1}$ then set N_i to N_{i-1}; the factor 15 was given by the maximum slope of the radon sensor output when Rn-charged water (i.e. of about 4700 Bq m^{-3} reaches the membrane-equilibration tube of the Rn-monitor (RTM-1688) with the given sampling configuration (fast mode, 5 min integration time), initial water in the equilibration tube of ~76 Bq m^{-3}, and pumped volume rate in the flow-through equilibration tube.[3]

3. If the ^{222}Rn activity concentration in air during decline after peak maximum is: $N_i > 1.7 x N_{i+1}$ then set N_i to N_{i-1}; this argument considers the physical radon decay, which implies that 59% (=1/1.7) of the initial Rn activity concentration is measured on average within a 5-min interval.

The filtered radon activity concentrations in air in the inner gas chamber of the RTM-1688 were subsequently converted into radon activity concentrations in water by multiplying all data with an Ostwald-coefficient of 0.17. The coefficient converts a radon activity concentration in air to a radon activity concentration in seawater at the given temperature and salinity during measurements (i.e. T_{mean}= 27.5°C; S_{mean}= 35.8). As a result of the applied filtering procedure, the majority of radon activity concentration in seawater were set to the measured local background of 4 Bq m^{-3} and a maximum of about 3100 Bq m^{-3} was determined during the monitoring campaign (Figure S 3-4).

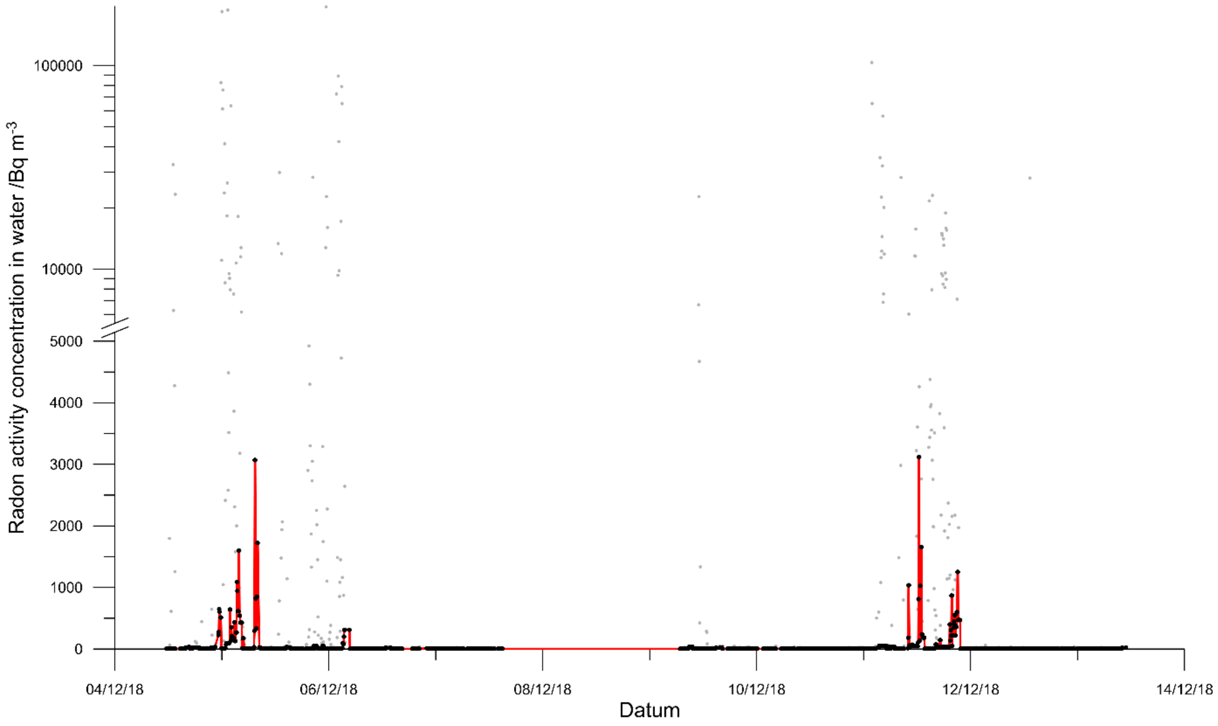

Figure S3-4. Radon activity concentration measured along the Wave glider track off Taqa. Black dots connected by red line represent the final Radon data after spike correction (all data in grey).

Gas ebullition

Ebullition (i.e., the spontaneous formation of gas bubbles in seawater) might strip ^{222}Rn out of the plume water. In order to evaluate the potential effect of ebullition on the field observations, we considered the case where the groundwater discharged at the seafloor would have been saturated with aqueously dissolved gases (such as CH_4 or CO_2). In this case, ebullition could happen during ascent in the water column due to decreasing pressure. Ebullition is controlled by two antagonistic processes happening during plume ascent: entrainment in the ascending plume of ambient water undersaturated with dissolved gases decreases the oversaturation in plume water, whereas decreasing ambient pressure increases the oversaturation, potentially leading to ebullition. We find

that ebullition is unlikely to have posed a major limitation in the current study owing to the dominant effect of dilution during most of the ascent through the water column. For the base case simulation, which predicted the volume of water entrained in the plume over its ascent trajectory, we evaluated the potential of ebullition for the conditions the most favorable to ebullition, i.e. if the discharged water would be 100% saturated with aqueously-dissolved gases and the ambient seawater would contain a total pressure of 1 atm of dissolved gases throughout the water column. Under these conditions and assuming Henry's law, water would initially become undersaturated with dissolved gases due to entrainment of ambient seawater in the plume. Upon nearing the water surface the pressure decrease would reduce the extent of undersaturation until water would become oversaturated with dissolved gases (i.e., the total pressure of dissolved gases would exceed the ambient pressure meaning that ebullition would be theoretically possible). However, the plume water would become oversaturated only within 3.3 m of the sea surface (7 s before reaching the sea surface), with a maximum oversaturation of dissolved gases of 0.14 atm. These simulated conditions are the most favorable conditions for ebullition, and field conditions are likely to have been less favorable to this process (groundwater less than 100% saturated with dissolved gases). Consequently, we interpret that ebullition is unlikely to have substantially affected the field measurements performed with the sensor mounted on the wave glider.

S-4 Model parameters

Table S4. Property values used in the TAMOC simulations.

Parameter	base case value	uncertainty range	comment
ambient seawater profiles of temperature and salinity	in-situ CTD measurement (Figure 4c, d)	-[a]	the CTD profile was acquired at a nearby location and at the same time of year, but on a different year (Figure 2)
cross current	0.27 m s^{-1}	- [a]	average of water current measured at the sea surface by the wave glider; assumed representative of the average situation within the water column by lack of additional measurements
volume flow rate of the groundwater discharge	9.8 m^3 s^{-1}	<0.2–100 m^3 s^{-1}	based on reported flow rates in the literature[4]
diameter of the (assumed circular) discharge zone of seafloor	5 m	5–15 m	base case value based on in-situ field observations
salinity of the discharged groundwater	13.5	4–23	mean (base case) and range of measured values at 3 nearby deep onshore wells
temperature of the discharged groundwater	30°C	28–32°C	mean (base case) and range of measured values at 3 nearby deep onshore wells
Radon activity concentration of the	50,000 Bq m^{-3}	25,000–100,000 Bq m^{-3}	Water from the 3 nearby deep onshore wells spanned the range 9,000–49,000

discharged groundwater			Bq m^{-3}; the radon activity concentration of the subsea discharge remains poorly constrained, and values >200,000 Bq m^{-3} have been reported in sediment and meta-sediment aquifers.[5] The selected range covers the likely range based on TAMOC simulations.
Water depth	100 m	- [a]	Approximate water depth at which most of the elevated radon activity concentrations were observed
velocity of the discharged water at the seafloor	1.0 m/s	0.0001–2.0 m s^{-1}	water velocity up to 2.0 m s^{-1} has been deduced for a karstic cave.[6]

[a] uncertainty range of the parameter not evaluated.

S-5 Bathymetry information

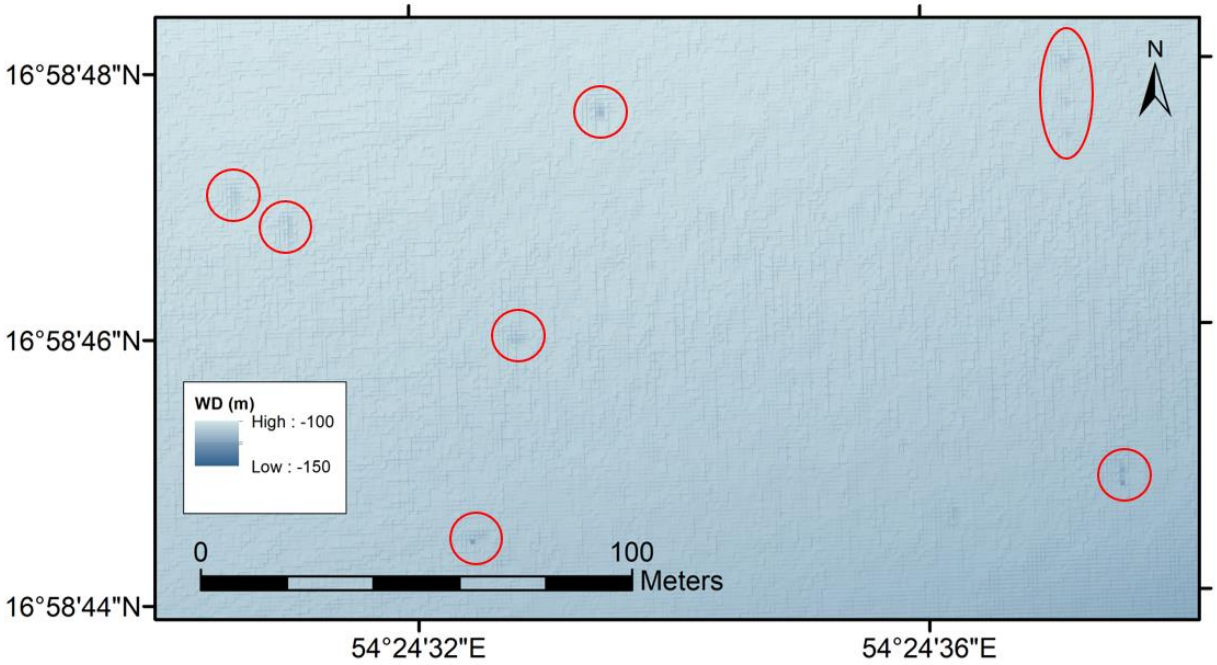

Figure S 5-1. Bathymetric map indicating small depressions (circled in red) at water depths of about 110 m (~ 6 km south of Taqa)

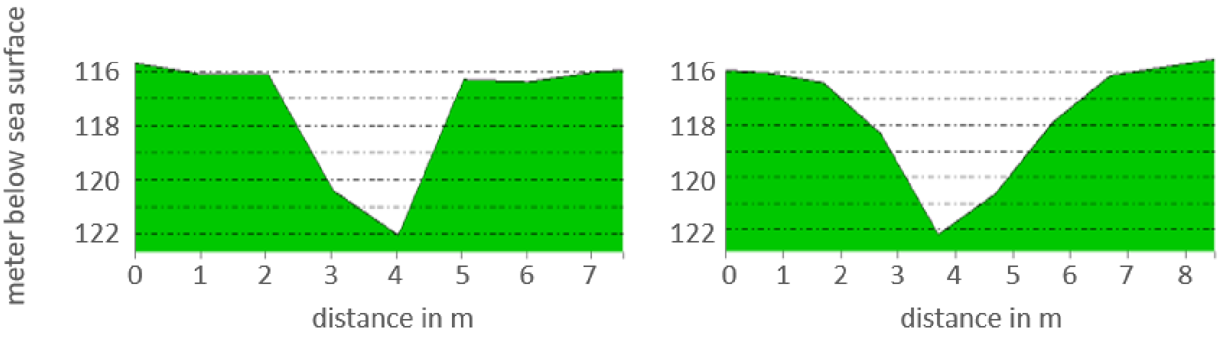

Figure S 5-2. The shape of a depression is illustrated by a south-north depth profile (left) and an east-west depth profile (right)

Table S 5. Measured dimensions of seafloor depressions located on the shelf area off Taqa. Water depths are given in meters below sea level (mbsl).

ID	Diameter (m)	Center (mbsl)	Rim (mbsl)
1	5	117.2	115.6
2	5	119.8	116.2
3	4	120.6	118.1
4	4	122.3	120.7
5	6	125.2	124.2
6	4	125.4	122.7
7	4	125.8	122.7
8	6	121.3	116.0
9	4	118.3	116.7
10	3	118.1	116.9

S-6 Catchment area Teyq sinkhole

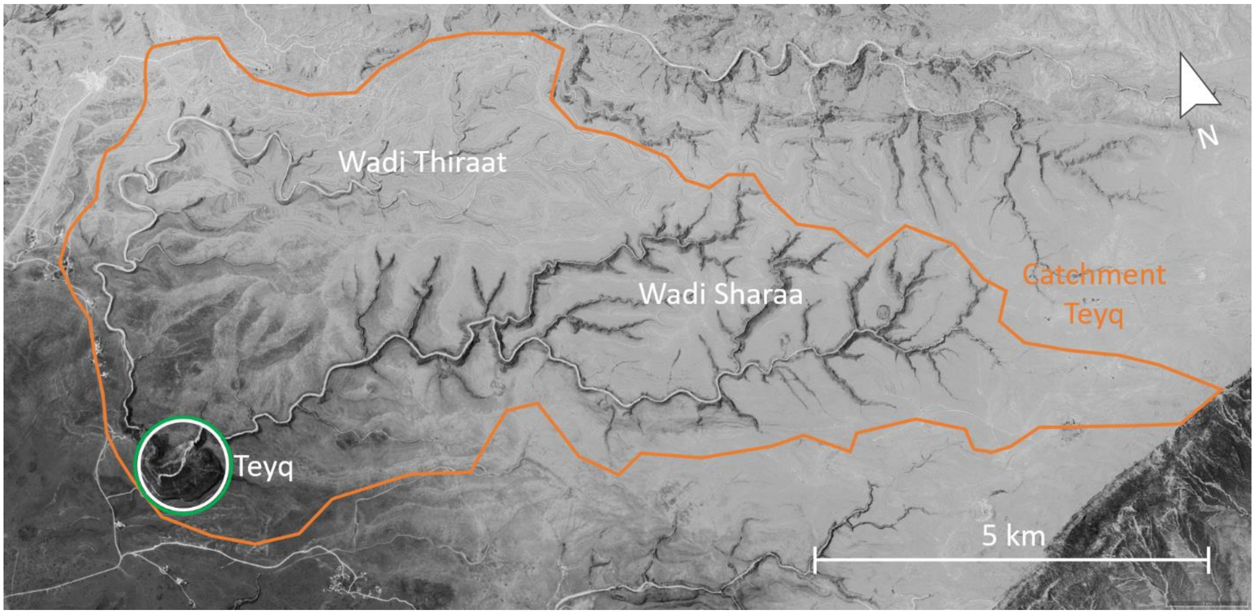

Figure S 6. Surface catchment area of the Teyq sinkhole is about 50km². For location see Figure 1. Catchment area was constructed using ArcGIS 10.6. Map imagery reprinted with permission from Earthstar Geographics. Copyright 2022 Earthstar Geographics/ Terracolor.

References

(1) Platel, J. P.; Qidwai, H. A.; Khalifa, M. I. Geological Map of Marbat, Sheet NE-40-9E, 1:100,000, with Explanatory Notes., 1987.

(2) Petermann, E.; Schubert, M. Quantification of the Response Delay of Mobile Radon-in-Air Detectors Applied for Detecting Short-Term Fluctuations of Radon-in-Water Concentrations. *Eur. Phys. J. Spec. Top.* **2015**, *224* (4), 697–707. https://doi.org/10.1140/epjst/e2015-02400-5.

(3) Al Balushi, H. *Detecting Submarine Groundwater Plumes in the Salalah Area: A Radon Tracer and Numerical Integral Plume Model*; Master Thesis; GEOMAR, 2019; p 70.

(4) Fleury, P.; Bakalowicz, M.; de Marsily, G. Submarine Springs and Coastal Karst Aquifers: A Review. *J. Hydrol.* **2007**, *339* (1), 79–92. https://doi.org/10.1016/j.jhydrol.2007.03.009.

(5) Girault, F.; Perrier, F.; Przylibski, T. A. Radon-222 and Radium-226 Occurrence in Water: A Review. *Geol. Soc. Lond. Spec. Publ.* **2018**, *451* (1), 131–154. https://doi.org/10.1144/SP451.3.

(6) Jeannin, P.-Y. Modeling Flow in Phreatic and Epiphreatic Karst Conduits in the Hölloch Cave (Muotatal, Switzerland). *Water Resour. Res.* **2001**, *37* (2), 191–200. https://doi.org/10.1029/2000WR900257.
